# Association between physical activity, socioeconomic status, blood biomarkers, and diet in lebanese adults

**DOI:** 10.1371/journal.pone.0321884

**Published:** 2025-04-25

**Authors:** Elie-Jacques Fares, Maria Abou Mourad, Marco Bardus, Sarah Zaki, Marie Claire Chamieh

**Affiliations:** 1 Nutrition and Food Sciences, American University of Beirut, Beirut, Lebanon; 2 Department of Medicine, Faculty of Medicine and Health Sciences, University of Sherbrooke, Canada; 3 Department of Applied Health Sciences, School of Health Sciences, College of Medicine and Health, University of Birmingham, Edgbaston, United Kingdom; Saint-Joseph University of Beirut, LEBANON

## Abstract

**Background:**

Inactivity is a significant contributor to non-communicable diseases. In Lebanon, the World Health Organization reported a rising prevalence of physical inactivity among adults. Various studies highlight the benefits of physical activity (PA) on health, influenced by sociodemographic factors, gender, age, and diet. This study aims to examine PA correlates in Lebanese adults, focusing on blood biomarkers.

**Methods:**

This cross-sectional study included 296 adults aged ≥18 years. Participants completed a brief sociodemographic and food frequency questionnaire, underwent anthropometric measurements, and provided fasting blood samples. PA was measured using the International Physical Activity Questionnaire (IPAQ) short form and was divided into two categories: low PA corresponding to any walking activity, and moderate to vigorous PA for activities requiring physical effort. Descriptive statistics were computed for sociodemographic characteristics, BMI, waist circumference, energy intake, PA levels, and blood biomarkers. Logistic regressions were used to assess PA and blood biomarkers associations, adjusted for relevant covariates.

**Results:**

Gender and marital status were associated with moderate to vigorous PA levels. No association was found between PA levels, BMI, waist circumference, diet, or blood biomarkers. Multivariate binary logistic regression analyses showed that females (OR=1.96, 95% CI: 1.16–3.31) and those with LDL moderate risk (OR=1.90, 95% CI: 1.02–3.66), and high risk (OR=2.44, 95% CI: 1.08–5.55), were more likely to show moderate-to-high PA levels.

**Conclusion:**

PA was positively associated with gender and disease risk, particularly LDL, a biomarker known to jeopardize cardiovascular health. Disease risk appears to be a driving factor in performing physical activity among women. These results may guide early nutrition interventions endorsing physical activity as a preventive measure to decrease the prevalence of cardio metabolic disorders.

## Introduction

Non-communicable diseases (NCDs), including cardiovascular diseases, cancer and diabetes, cause 41 million deaths annually worldwide [1]. Inactivity is a major risk factor for NCDs [2,3] contributing to elevated blood pressure, cholesterol levels, glycemia, and obesity [1,4,5]. Concomitantly, studies have shown that Physical Activity (PA) influences some health indicators such as blood biomarkers and BMI [6,7].

Different factors, such as sex, age, diet, BMI and Socioeconomic status (SES), affect PA [8–10]. According to WHO, men perform more PA than women [11]. However, women have been reported to have substantially higher household PA levels compared to men [12]. Numerous studies have reported a negative relationship between age and PA for both genders [13–15]

In 2022, the WHO reported that the prevalence of physical inactivity among Lebanese adults was 40% in males and 33% in females [11]. Earlier in 2015, research revealed an obesity prevalence of 26.1% in Lebanese adults with a significant negative association between BMI and PA, particularly among women [16]. Research documents a negative correlation between BMI and PA [17]. Likewise, several studies indicate that individuals with higher BMI are more likely to have sedentary lifestyles [4,18–20], as well as altered blood biomarkers manifested as aberrant lipid profile [21].

Diet also significantly influences PA. Simoes et al. reported that higher leisure-time PA leads to decreased dietary fat consumption [22]. Gillman et al. showed that sedentary adults consumed less healthy foods, such as fruits and vegetables, and fewer nutrients (calcium, folate, vitamins A, C, and E) than active participants [23]. In Saudi Arabia, approximately 80% of adults eat fast food at least once per week [24] and more than 70% of adults are physically inactive [25]. Studies in Spain and Italy suggest that adherence to the Mediterranean diet correlates with higher PA levels [26,27]. Although positive association was shown between PA and healthy diet, few studies have examined dietary behavior [8].

Socioeconomic status (SES) is another key determinant of PA [10]. Higher SES reflected through income, education, and occupation [28], is positively associated with PA [15,29]. Several studies have reported that people with a higher SES are more likely to participate in PA than those with a lower SES [9]. For example, a study investigating socioeconomic differences in sports and PA among Italian adults showed that highly educated participants were more likely to be physically active than their less-educated counterparts [30]. Conversely, occupation was negatively associated with PA.

While many studies have focused on consistent correlates of PA such as age, sex, marital status, and SES [31], fewer have addressed these associations in Lebanon [20]. Research indicates a higher prevalence of metabolic syndrome among older individuals [32], often related to changes in dietary habits [33]. It would be thus interesting to additionally examine the association between blood biomarkers and PA, particularly among Lebanese adults. This study assessed the demographic, dietary, socioeconomic, and blood biomarker determinants of PA in Lebanese adults and estimated its prevalence. We hypothesized that individuals with normal blood biomarker levels are more physically active.

## Materials and methods

### Study design

The current study is a secondary analysis of the National WHO Stepwise survey, “Nutrition and Noncommunicable Diseases Risk factors cross-sectional survey,” conducted in Lebanon between 2008 and 2009 [34]. The study was conducted with a nationally representative sample of Lebanese individuals aged > 6 years.

### Setting

Study samples were drawn from randomly selected households, based on stratified cluster sampling: the strata were the Lebanese Governorates, the clusters were selected further at the level of districts, urban and rural areas, and the housing units constituted the primary sampling units in the different districts of Lebanon. One adult from each household and one child/adolescent from every other household were selected from the household roster, excluding pregnant and lactating women and subjects with mental disabilities. Field work was carried between May 2008 and August 2009 with a final sample including 3636 [33]. Participants aged 18–65 years with no chronic diseases (n=1,331) were then contacted to provide blood sample. Further details regarding the design of the original survey can be found in Naja et al. [35].

### Participants

For this study, data of the 323 subjects that provided a blood sample (response rate: 25.2%), was used. After data cleaning, the age group was determined to be between 18 and 65 years old. The final sample size was (n=296). This study was conducted in accordance with the Declaration of Helsinki guidelines. The original study design and protocol were approved by the Institutional Review Board (IRB) of the American University of Beirut (Protocol Number: FHS.AS.04), and informed consent was obtained from all participants.

### Variables

#### Physical Activity.

The Arabic translation of the short form of the IPAQ was used to interview participants [33]. It has been validated and used in health-related studies in the MENA region [16,36–38]. It includes questions on demographics, household characteristics, smoking, dietary intake, PA, and reported diseases. The short form of the IPAQ comprises seven items that provide information on the time spent walking, vigorous- and moderate-intensity physical activity, and sedentary activity [39]. It measures total PA in all settings, including transportation, work, and leisure time [40]. The total PA was calculated by weighing each type of activity by its energy requirements defined in METs (multiples of the resting metabolic rate: walking = 3.3 METs; moderate intensity = 4.0; METs, vigorous intensity = 8.0 METs). This was conducted to yield an MET minutes/week score, which was later categorized into three activity levels: low, moderate, and high. A low PA corresponds to any walking activity. Moderate PA refers to activities that require moderate physical effort and cause participants to breathe harder than normal. High PA corresponds to activities that require hard physical effort and make people breathe much harder than normal [41].

#### Diet.

A 61-item Food Frequency Questionnaire (FFQ) was used to measure food intake over the past year. Daily gram intake of food items, energy, and macronutrient intake was computed using the food composition database of Nutritionist IV software (Nutritionist IV program, 1997, First Databank Inc., San Bruno, CA, USA). A trained dietitian administered the FFQ.

#### Blood Biomarkers.

Blood biomarker data (triglycerides, HDL, LDL, VLDL, and CRP) were obtained from the original study and analyzed. Blood samples were collected after an overnight fast. The serum was centrifuged and shipped on dry ice to the AUB Laboratory. The levels of triglycerides, HDL-C, and glucose were measured using an enzymatic spectrophotometric method with a Vitros 350 analyzer (Ortho Clinical Diagnostics, Johnson & Johnson, 50–100 Holmers Farm Way, High Wycombe, Buckinghamshire, HP12 4DP, United Kingdom). The inter-assay variation in the measurements did not exceed 4%. Quality control was performed within each run by using standard performance verifier solutions provided by Ortho Clinical Diagnostics. All samples were analyzed in duplicate, and the average value was used for statistical analysis [35].

#### Data Treatment Guidelines.

For Data Processing and Analysis of the IPAQ, the following guidelines were followed [41];Total PA: Each-type of activity was defined in METs to yield a score in MET-minutes/week, which was later categorized into three levels of activity: low, moderate, and high [41]. The dependent variable “PA” was dichotomized into two categories to run the binary logistic regression: low PA level (reference category), which corresponds to negligible physical activities (e.g., walking), and moderate-to-vigorous PA level, which corresponds to a minimal level of physical activity and health-enhancing activities. Continuous variables, such as HDL, LDL, VLDL, and CRP levels, were categorized according to the risk cut-off. Age was categorized according to its frequency in the dataset.

#### Study Sample size.

To ensure the adequacy of our sample size for detecting meaningful associations between physical activity (PA) levels and blood biomarkers, we conducted a power analysis. Using the G*Power software [42], a well-regarded statistical power analysis tool, we estimated the required sample size based on the following parameters: moderate effect size for logistic regression (OR=1.56), significance level (α) of 0.05, which is standard for maintaining a balance between Type I and Type II errors, and power (β) ranging between 0.8 and 0.95, indicative of an 80–95% probability of correctly rejecting a false null hypothesis. Our power analysis indicated that a sample size between 206 and 334 participants would be necessary under these parameters.

#### Statistical Analysis.

Statistical analysis was performed using the IBM SPSS Statistics ver. 20.0 (IBM Co., Armonk, NY, USA). It was used for data analysis and to determine the association between the dependent variable (PA) and independent variables (diet, blood biomarkers, and SES). Sociodemographic, anthropometric, lifestyle, and clinical characteristics are presented as frequencies, means, and standard deviations (SD). The associations between two categorical variables were determined using the chi-square test. Energy intake and percentage of macronutrient consumption are presented as mean ± standard deviation (mean ± SD). A binary logistic regression was conducted with PA as the dependent variable and age, gender, marital status, education, crowding index, waist circumference, and LDL levels as covariates. Odds ratios (ORs) were calculated using 95% confidence intervals (CI). Statistical significance was set at p < 0.05.

## Results

A total of 138 men (46.6%) and 158 women (53.4%) aged 18–65 years participated in this study. Sociodemographic characteristics, blood biomarkers, dietary measures, and physical activity of the study sample are shown in Table 1. Of the participants, 60.1% were married, 55.1% were self-employed or employed, 58% were overweight or obese, 66.6% had a crowding index ≥1 person/room, and 65.2% had moderate-to-vigorous intensity activity. The mean energy intake was 2702.5 Kcal/day, of which 48.1% was derived from carbohydrate consumption. Mean values of HDL, LDL, VLDL, CRP were 51.3 mg/dL, 129.4 mg/dL, 26.9 mg/dL, and 4.9 mg/L respectively.

**Table 1:** Demographic characteristics, blood biomarkers, dietary measures, and physical activity in the study sample (n=296).

Demographics	N(%)
**Age groups (years)**	
18.0-39.9	199(67.2)
40.0-65.0	97(32.8)
**Gender**	
Men	138(46.6)
Women	158(53.4)
**Marital Status**	
Not married	118(39.9)
Married	178(60.1)
**Work Status**	
Worker	163(55.1)
Not working	133(44.9)
**Crowding Index**	
CI<1 person/room	97(32.8)
CI≥1 person/room	197(66.6)
**Physical activity**	
Low Intensity Activity	103(34.8)
Moderate/Vigorous Activity	193(65.2)
**Anthropometrics**	**N(%)**
**Body mass index (Kg/m2)**	
Underweight or Normal weight	123(42)
Overweight/Obese	170(58)
**Waist Circumference**	
Normal	146(49.3)
High	150(50.7)
**Blood Biomarkers**	**Mean (±SD)**
HDL (mg/dL)	51.3(**±** 14.7)
LDL (mg/dL)	129.4(**±** 38.9)
VLDL (mg/dL)	26.9(**±** 14.6)
CRP (mg/L)	4.9(**±** 6.8)
Glucose (mg/dL)	102(**±** 22.2)
25OHD (ng/mL)	15.4(**±** 7.1)
Insulin (μLU/mL)	23.2 (**±** 17.1)
**Dietary Intake**	**Mean (±SD)**
**Caloric intake (Kcal/day)**	2702.5(**±** 1145.3)
**% Calories from carbs**	48.1(**±** 6.8)
**% Calories from proteins**	15.6(**±** 3)
**% Calories from fat**	36.4(**±** 6.8)

Crowding index = number of persons in the household divided by the number of rooms, excluding kitchen & bathrooms.

The sociodemographic characteristics stratified by PA level (low, moderate-to- vigorous) are presented in Table 2. There was a significant difference in PA between males and females (p =0.003), whereby moderate-to-vigorous PA was more prevalent in females than in males (59.6% vs. 40.4%, respectively). In addition, there was a significant relationship between marital status and PA (p = 0.006), whereby married participants were more likely to engage in moderate-to-vigorous PA than unmarried participants.

**Table 2:** Distribution of baseline characteristics among Lebanese adults (n=296) according to levels of physical activity.

Variables	Physical activity levels		
	Low	Moderate/ Vigorous	*p*-value
	(n= 103)	(n=193)	
**Categorical Variables**	**N%**		
**Age groups (years)**			
18.0-39.9	73(70.9)	126(65.3)	.329
40.0-65	30(29.1)	67(34.7)	
**Gender** ^*^	43(41.7)		
Men	60(58.3)	78(40.4)	**.003**
Women		115(59.6)	
**Marital Status** ^*^			
Not married	52(50.5)	66(34.2)	
Married	51(49.5)	127(65.8)	**.006**
**Educational level**			
Complementary or less	39(37.9)	71(36.8)	
Secondary or Technical	27(26.2)	64(33.2)	.407
University	37(35.9)	58(30.1)	
**Work Status**			
Worker	64(62.1)	99(51.3)	.074
Not working	39(37.9)	94(48.7)	
**Crowding Index**			
CI <1 person/room	36(35)	61(31.9)	.600
CI ≥1 person/room	67(65.0)	130(68.1)	
**Body mass index (Kg/m**^**2**^)			
Underweight/Normal	48(47.1)	75(39.3)	
Overweight/Obese	54(52.9)	116(60.7)	.198
**Waist Circumference**			
Normal	57(55.3)	89(46.1)	.130
Elevated WC	46(44.7)	104(53.9)	

*Significant at P<0.05. P-Values were derived from chi-square test for all socio demographic distributions and health characteristics.

The blood biomarker levels stratified by PA levels among the Lebanese adults (n=296) are presented in Table 3. The only significant association was found between PA and LDL levels (p =0.025). Those at moderate risk had a high prevalence of moderate-to-vigorous PA.

**Table 3:** Blood biomarkers values among Lebanese adults (n=296) according to levels of physical activity.

Blood Biomarkers	Physical activity levels			
	Total	Low	Moderate/ Vigorous	*p*-value
	(n=296)	(n= 103)	(n=193)	
**Categorical Variables**	**N%**			
**HDL male (mg/dL)**				
Normal > 40	81(58.7)	34(56.7)	47(60.3)	.671
At risk ≤ 40	57(41.3)	26(43.3)	31(39.7)	
**HDL female (mg/dL)**				
Normal > 50	114(72.2)	32(74.4)	82(71.3)	.698
At risk ≤ 50	44(27.8)	11(25.6)	33(28.7)	
**LDL (mg/dL)** ^*^				
Normal: ≤ 100	64(21.7)	31(30.4)	33(17.1)	
Moderate risk: 101–159	169(57.3)	54(52.9)	115(59.6)	**.025**
High risk: ≥ 160	62(21)	17(16.7)	45(23.3)	
**VLDL (mg/dL)**				
Desirable < 30	204(69.2)	68(66.7)	136(70.5)	.502
At risk ≥ 30	91(30.8)	34(33.3)	57(29.5)	
**CRP (mg/L)**				
Low risk CVD: <1	25(8.5)	9(8.9)	16(8.3)	
Intermediate risk: 1–3	124(42.3)	39(38.6)	85(44.3)	.646
High risk: > 3	144(49.1)	53(52.5)	91(47.4)	
**Glucose (mg/L)**				
Normal: 70–99	172(58.1)	65(63.1)	107(55.4)	.203
Insulin resistance: ≥100	124(41.9)	38(36.9)	86(44.6)	
**25OHD (ng/mL)**				
Normal: 30–100	7(2.9)	2(2.6)	5(3.0)	
Insufficiency:20–30	54(22.2)	13(16.9)	41(24.7)	.377
Deficiency: < 20	182(74.9)	62(80.5)	120(72.3)	
**Insulin (μIU/mL)**				
Normal: 6–27	184(77.6)	57(77)	127(77.9)	.879
Hyperinsulinemia > 27	53(22.4)	17(23)	36(22.1)	

*Significant at P<0.05. P-Values were derived from chi-square test for all blood biomarkers values.

Potential independent variables and covariates of theoretical importance were explored individually in a simple logistic regression and were shown in Table 4. The results revealed significant associations between gender, marital status, and LDL cholesterol levels with moderate-to-high intensity PA levels. Women were significantly more likely to engage in moderate-to-high PA than men (OR = 2.057, 95% CI: 1.266–3.344, p = 0.004). Similarly, the odds for married individuals to engage in moderate-to-vigorous PA were higher than those who were single, divorced, or widowed (OR = 1.962, 95% CI: 1.205–3.195, p = 0.007). Additionally, individuals with moderate (101–159 mg/dL) and high (≥ 160 mg/dL) LDL levels tended to engage more in PA (OR = 2.001, 95% CI: 1.112–3.599, p = 0.021; OR = 2.487, 95% CI: 1.183–5.226, p = 0.016, respectively). However, no significant associations were found between PA levels and other variables, including age, education level, BMI, waist circumference, work status, HDL, VLDL, CRP, glucose, 25OHD, and insulin (p > 0.05).

**Table 4:** Odds ratio estimates and their 95% confidence intervals from simple logistic regression analysis for the association of socio-demographic characteristics and blood biomarkers with moderate-to-high intensity activity among study participants.

	95% C l for OR			
Variables	OR	Lower	Upper	p-value
**Age groups (years)**				
18.0-39.9				
40.0-65	1.294	.771	2.172	.330
**Gender**				
Men				
Women	2.057	1.266	3.344	**.004**
**Marital Status**				
Single/Divorced/Widowed	1.00	1.00	1.00	
Married	1.962	1.205	3.195	**.007**
**Educational level**				
Complementary or less	1.00	1.00	1.00	
Secondary or Technical	1.302	.718	2.362	.385
University	.861	.488	1.520	.606
**Crowding Index**				
CI <1 person/room	1.00	1.00	1.00	
CI ≥1 person/room	1.145	.690	1.900	.600
**Waist Circumference (WC)**				
Normal	1.00	1.00	1.00	
Elevated WC	1.448	.895	2.342	.131
**Work Status**				
Worker	1.00	1.00	1.00	
Not working	1.558	.956	2.538	.075
**Body mass index**				
Underweight or Normal weight	1.00	1.00	1.00	
Overweight/Obese	1.375	.846	2.234	.199
**HDL male (mg/dL)**				
Normal > 40	1.00	1.00	1.00	
At risk ≤ 40	.863	.436	1.707	.671
**HDL female (mg/dL)**				
Normal > 50	1.00	1.00	1.00	
At risk ≤ 50	1.171	.529	2.593	.698
**LDL (mg/dL)**				
Normal: ≤ 100	1.00	1.00	1.00	
Moderate risk: 101–159	2.001	1.112	3.599	**.021**
High risk: ≥ 160	2.487	1.183	5.226	.**016**
**VLDL (mg/dL)**				
Desirable < 30	1.00	1.00	1.00	
At risk ≥ 30	.838	.501	1.403	.502
**CRP (mg/L)**				
Low risk CVD: <1	1.00	1.00	1.00	
Intermediate risk: 1–3	1.226	.498	3.016	.657
High risk: > 3	.966	.399	2.338	.939
**Glucose (mg/L)**				
Normal: 70–99	1.00	1.00	1.00	
Insulin resistance: ≥100	1.375	.842	2.246	.204
**25OHD (ng/mL)**				
Normal: 30–100	1.00	1.00	1.00	
Insufficiency:20–30	1.262	.218	7.292	.795
Deficiency: < 20	.774	.146	4.105	.764
**Insulin (μIU/mL)**				
Normal: 6–27	1.052	.546	2.028	.879
Hyperinsulinemia > 27	1.00	1.00	1.00	

For sociodemographic characteristics and blood biomarkers, the OR and 95% confidence intervals for their association with moderate-to-high-intensity activity are shown in Table 5. The results showed that being married with moderate-risk LDL values increased the odds of women performing moderate-to-vigorous PA. Although not statistically significant, work status was associated with higher odds of PA in those who were not working.

**Table 5:** Odds ratio estimates and their 95% confidence intervals from multivariate logistic regression analysis for the association of socio-demographic characteristics and blood biomarkers with moderate-to-high intensity activity among study participants.

	95% C l for OR			
Variables	OR	Lower	Upper	p-value
**Age groups (years)**				
18.0-39.9				
40.0-65	.864	.472	1.580	.635
**Gender**				
Men				
Women	1.964	1.166	3.308	**.011**
**Marital Status**				
Single/Divorced/Widowed	1.00	1.00	1.00	
Married	1.415	0.792	2.528	.241
**Educational level**				
Complementary or less	1.00	1.00	1.00	
Secondary or Technical	1.381	.736	2.590	.814
University	.928	.498	1.730	.230
**Crowding Index**				
CI <1 person/room	1.00	1.00	1.00	
CI ≥1 person/room	.937	.545	1.613	.815
**Waist Circumference (WC)**				
Normal	1.00	1.00	1.00	
Elevated WC	1.121	.658	1.910	.675
**LDL (mg/dL)**				
Normal: ≤ 100	1.00	1.00	1.00	
Moderate risk: 101–159	1.944	1.032	3.659	**.040**
High risk: ≥ 160	2.442	1.075	5.548	.**033**

Multivariate logistic regression stratified by physical activity levels and adjusted for all the variables included in the model

## Discussion

This study aimed to estimate the associations between sociodemographic factors, anthropometric covariates, and other biomarkers with physical activity. Compared to another similar cross-sectional survey [16], physical activity was associated with gender, but not with other sociodemographic factors. It is known that physical activity levels varies by various factors (sex, age, socioeconomic factors, etc …) [43–45] and among individuals according to lifestyle habits, personal preferences, and health conditions [46].

These results are in line with those reported in a Swedish cross-sectional study in which 63% of adult participants exhibited moderate to high levels of physical activity [47]. Additionally, the prevalence of physical activity observed in this study exceeded that reported in 2009 by Tannir et al., which identified 55.5% of Lebanese participants as being physically active [20]. However, it is lower than another cross-sectional study performed in Morocco, in which 83.5% of adult participants demonstrated moderate to high levels of physical activity [48]. This might be due to the cross-sectional nature of the study, involving volunteers, albeit recruited from a nationally representative sample.

This study did not show significant associations with dietary indicators, despite the evidence from reviews and primary studies.

In recent years, substantial shifts have been witnessed in the lifestyle practices of adults in Lebanon, propelled by various economic, social, and political crises that affected their daily lives. Lebanon is a small, middle-income country in the Mediterranean Sea that renders the health of its population a complex system. A study conducted by Naja et al. revealed that, even with a high load of fruits and vegetables in the Lebanese pattern, high-calorie dairy products and traditional sweets are heavily consumed. This may counteract the benefits of fruits and vegetables and contribute to a higher risk of metabolic syndrome [35].

Furthermore, research conducted by Naja et al. in 2011 identified four Lebanese dietary patterns: Western, traditional, prudent, and fish and alcohol [48]. The findings demonstrated that women adhered more to the prudent dietary pattern, whereas men adhered more to the Western pattern. This trend aligns with other observations in literature where adherence to the prudent pattern was positively associated with education. This could be explained by the fact that education enables people to make informed choices about their health [49].

This study found that moderate-to-vigorous physical activity was higher in married (65.8%) than in unmarried individuals (34.2%). Empirical evidence suggests that marriage can positively influence physical activity levels in both men and women. Furthermore, marriage has been shown to strongly impact the decision to participate in physical activities [50]. This could be due to the social support and encouragement provided by a spouse or partner, as well as the shared interests and activities that often come with a committed relationship. This was reported in a previous study by Polish researchers that revealed two major results. Having a partner or spouse may have a positive effect on the quality of life of female students [51]. Furthermore, a 2-year prospective analysis performed by Hull et al. reported that having a child significantly reduces parents’ PA levels, with no impact from marriage, with feelings of exhaustion and overwhelm being frequently reported by new parents [52]. Our analyses focused solely on marital status, with no analysis of the other significant factors.

However, other studies have shown mixed results regarding the association between marriage and PA. A cross-sectional study in Poland showed that single participants were more likely to meet the WHO recommendations for PA than married participants [53]. Another study reported that married men’s PA levels were related to their wives’ PA participation. Thus, actively married men are three times more likely to have active wives [54].

This study also revealed that women (53.4%) were more likely to engage in physical activity than men (46.6%). Therefore, married women may be more likely than married men to prioritize physical activity to maintain health and well-being, especially as they progress with age. Moreover, women tend to have more household and childcare responsibilities, which may require them to be more active throughout the day. A similar observation was reported in a study investigating the distribution of housework among multiple couples in different European countries. A higher level of leisure-time physical activity has been reported among women, who are responsible for a greater load of household chores [55].

Employed individuals exhibit higher overall quality of life and satisfaction with their health [51]. These findings were further confirmed in the present study. Although not statistically significant, this study underscored that those who did not work were less likely to engage in PA. Employment increases the likelihood of fostering consistency and establishing a structured daily routine. This is essential to sustain regular physical activity. Additionally, going out to work rather than staying home-bound contributes to higher energy levels and enthusiasm for PA. It is worth noting that work can create a stressful environment that requires more enjoyable physical activities to compensate for the daily social burden.

This study showed that individuals with a moderate LDL risk tended to engage in more physical activity. LDL cholesterol is often known to be the “bad” cholesterol because it can build up in the walls of arteries, leading to atherosclerosis and an increased risk of heart disease [56]. PA fosters cardiovascular health and improves lipid profiles, notably enhancing HDL cholesterol levels [57]. People with high LDL levels may increase their physical activity to lower their LDL cholesterol levels. This could be considered a compensatory mechanism initiated by the body in response to the risk associated with increased LDL. It is plausible that inflammation has not yet begun in subjects with a moderate LDL risk. This may facilitate initiation and sustained adherence to routine physical activity compared to those with a high LDL risk profile.

LDL cholesterol can be used as an indicator of cardiovascular disease [58]. Research has shown that the prevalence of heart disease increases proportionally with age and is higher in men than in women. Therefore, older men tend to have higher levels of LDL cholesterol [59]. This study showed that subjects at high LDL levels were more likely to have lower PA levels than those at moderate LDL risk. This indicates that older men with higher LDL levels had lower PA. This could be explained by the inverse relationship between age and ability to perform PA. According to Singh et al., aging significantly increases the risk for high blood levels of low-grade inflammatory markers. LDL-C is a major inducer of inflammation in the body and multiple (auto-) inflammatory diseases are potentially influenced by LDL-C through inflammasome activation [60].

In this study, CRP levels were tested in participants, and the results did not reveal a discernible association with PA. CRP is an acute-phase reactant protein that is not a marker of chronic inflammation [61]. CRP is released quickly at the start of infections or inflammatory conditions. For example, alpha-1-acid glycoprotein (AGP) rises more slowly and stays longer than CRP and can be used with CRP to determine the stage and severity of inflammation. To accurately analyze micronutrients biomarkers, the World Health Organization advises measuring AGP and CRP levels [62]. Another limitation of this study was that the CRP level was insufficient to assess inflammation.

Hence, the current study shows that high-risk LDL, which increases inflammation, may reduce PA. Unless supported by a professional, a person with high inflammation may have major difficulty in starting an exercise routine or any household task. This finding was confirmed by a large population-based cohort study [63,64]. These studies consistently showed an inverse association between markers of systemic inflammation and physical activity or fitness status, and data from several small-scale interventional studies support that exercise training lowers inflammation [64]. It seems like a vicious cycle; heightened inflammation lowers PA, subsequently exacerbating inflammation levels even further.

Unfortunately, although the assessments employed in this study are convenient because of their brevity, they are less sensitive than more comprehensive instruments in measuring PA. Moreover, the inclusion of additional markers, such as AGP, and consideration of parental status would contribute to a more comprehensive and refined analysis. The interaction of physical activity with all of the above-mentioned factors is a very complex system to investigate. Self-reporting is a major pitfall in such studies. It is subjective and governed by each subject’s knowledge and experience.

According to this study, being married supports PA initiation and consistency, particularly when a partner is involved in an exercise routine. Work status is a major determinant of psychological and emotional stability. Although some work environments may be a burden on participants’ mental status, they may have a positive impact on PA. In addition, notable disparities in PA between males and females were observed; females were more involved in moderate to vigorous activity. This is largely influenced by demand for daily housework.

In conclusion, this study demonstrated a positive association between PA, sex, and disease risk, mainly a biomarker known to jeopardize cardiovascular health. Disease risk appears to be a driving factor in performing physical activity, especially among women. These results may guide early nutritional interventions that endorse physical activity as a preventive measure to reduce the prevalence of cardiometabolic disorders.

## Supporting Information

S1 Data Deidentified Data Set (Excel Format). Legend: This file contains the raw data collected in Excel format .
(XLSX)

S2 Data Deidentified Data Set (SPSS Format). Legend: This file contains the same raw data in SPSS format, for statistical analysis and replication of findings. (SAV)
